# Supplementary material for: SMC condensin entraps chromosomal DNA by an ATP hydrolysis dependent loading mechanism in Bacillus subtilis
Source: eLife. 2015 May 7;4:e06659. doi: 10.7554/eLife.06659 (PMC4442127; doi:10.7554/eLife.06659)
Supplement: Supplementary file 1. — Genotypes of Bacillus subtilis strains. All strains are derivatives of either Bacillus subtilis 1A700 (Bacillus Genetic Stock Centre) or Bacillus subtilis 168 ED. DOI: http://dx.doi.org/10.7554/eLife.06659.016 [file elife06659s001.docx]

**Supplementary File 1**: Genotypes of *Bacillus subtilis* strains. All strains are derived from *Bacillus subtilis* 1A700 (Bacillus Genetic Stock Centre) or from *Bacillus subtilis* 168 ED (Domínguez-Cuevas et al., 2012).

| Name | Genotype |
| --- | --- |
| BSG1001 | *1A700, trpC2* |
| BSG1002 | *1A700, smc ftsY::ermB, trpC2* |
| BSG1007 | *1A700, Δsmc ftsY::ermB, trpC2* |
| BSG1008 | *1A700, smc(E1118Q) ftsY::ermB, trpC2* |
| BSG1045 | *1A700, smc(K37I) ftsY::ermB, trpC2* |
| BSG1046 | *1A700, smc(S1090R) ftsY::ermB, trpC2* |
| BSG1047 | *1A700, smc(D1117A) ftsY::ermB, trpC2* |
| BSG1104 | *1A700, smc-TEV-AviTag ftsY::(ermB birA), cat::scpA scpB, trpC2* |
| BSG1105 | *1A700, smc(S19C, R558C, N634C, R1032C)-TEV-AviTag ftsY::(ermB, birA), cat::scpA(E52C, H235C), trpC2* |
| BSG1107 | *1A700, smc-TEV-AviTag ftsY::tetL, cat::scpA scpB, trpC2* |
| BSG1108 | *1A700, smc(S19C, R558C, N634C, R1032C)-TEV-AviTag ftsY::tetL, cat::scpA(E52C, H235C), trpC2* |
| BSG1449 | *1A700, dnaN-HISx12-HaloTag::specR, trpC2* |
| BSG1459 | *1A700, dnaN(N114C, V313C)-HISx12-HaloTag::specR, trpC* |
| BSG1494 | *1A700, smc(S19C, R558C, N634C, R1032C)-TEV-HaloTag ftsY::ermB, cat::scpA(E52C, H235C), trpC2* |
| BSG1743 | *1A700, smc(S19C, R643C, R1032C)-TEV-HaloTag ftsY::ermB, cat::scpA(E52C, H235C), trpC2* |
| BSG1782 | *1A700, smc(S19C, R558C, N634C, R1032C)-TEV-HaloTag ftsY::ermB, cat::scpA(E52C, H235C), dnaN(N114C, V313C)::specR, trpC2* |
| BSG1783 | *1A700, smc(S19C, R558C, N634C, R1032C)-TEV-HaloTag ftsY::ermB, cat::scpA(E52C, H235C), ΔparB::kanR, dnaN(N114C, V313C)::specR, trpC2* |
| BSG1784 | *1A700, smc(S19C, K37I, R558C, N634C, R1032C)-TEV-HaloTag ftsY::ermB, cat::scpA(E52C, H235C), dnaN(N114C, V313C)::specR, trpC2* |
| BSG1785 | *1A700, smc(S19C, R558C, N634C, S1090R, R1032C)-TEV-HaloTag ftsY::ermB, cat::scpA(E52C, H235C), dnaN(N114C, V313C)::specR, trpC2* |
| BSG1786 | *1A700, smc(S19C, R558C, N634C, R1032C, E1118Q)-TEV-HaloTag ftsY::ermB, cat::scpA(E52C, H235C), dnaN(N114C, V313C)::specR, trpC2* |
| BSG1807 | *1A700, smc(S19C, R558C, N634C, R1032C)-TEV-HaloTag ftsY::ermB, cat::scpA(E52C, H235C), dnaN(N114C, V313C)::specR, trpC2* |
| BSG1809 | *1A700, smc(S19C, R558C, N634C)-TEV-HaloTag ftsY::ermB, cat::scpA(E52C, H235C), dnaN(N114C, V313C)::specR, trpC2* |
| BSG1810 | *1A700, smc(S19C, N634C, R1032C)-TEV-HaloTag ftsY::ermB, cat::scpA(E52C, H235C), dnaN(N114C, V313C)::specR, trpC2* |
| BSG1811 | *1A700, smc(S19C, R558C, N634C, R1032C)-TEV-HaloTag ftsY::ermB, cat::scpA(E52C), dnaN(N114C, V313C)::specR, trpC2* |
| BSG1812 | *1A700, smc(S19C, R558C, R1032C)-TEV-HaloTag ftsY::ermB, cat::scpA(E52C, H235C),dnaN(N114C, V313C)::specR, trpC2* |
| BSG1813 | *1A700, smc(S19C, R558C, N634C, R1032C)-TEV-HaloTag ftsY::ermB, cat::scpA(H235C), dnaN(N114C, V313C)::specR, trpC2* |
| BSG1831 | *1A700, smc(R558C, N634C, R1032C)-TEV-HaloTag-ftsY::ermB; camR::scpA(E52C, H235C), dnaN(N114C, V313C)::specR, trpC2* |
| BSG1832 | *1A700, smc(S19C, R558C, N634C, R1032C)-HaloTag ftsY::ermB, camR::scpA(E52C, TEV, H235C), dnaN(N114C, V313C)::specR, trpC2* |
| BSG1850 | *1A700, smc(S19C, R558C, N634C, R1032C)-TEV-HaloTag ftsY::ermB, camR::scpA(E52C, H235C) ΔscpB, dnaN(N114C, V313C)::specR, trpC2* |
| BSG1960 | *1A700, smc(S19C, R558C, N634C, R1032C)-TEV-HaloTag ftsY::ermB, cat::scpA(E52C, H235C), dnaN(N114C, V313C)::specR, parB(G77S)::kanR, trpC2* |
| BSG1961 | *1A700, smc(S19C, R558C, N634C, R1032C)-TEV-HaloTag ftsY::ermB, cat::scpA(E52C, H235C), dnaN(N114C, V313C)::specR, parB(R80A)::kanR, trpC2* |
| BSG1962 | *1A700, smc(S19C, R558C, N634C, R1032C)-TEV-HaloTag ftsY::ermB, cat::scpA(E52C, H235C), dnaN(N114C, V313C)::specR, parB(R149G)::kanR, trpC2* |
| BSG1963 | *1A700, smc(S19C, R558C, N634C, R1032C)-TEV-HaloTag ftsY::ermB, cat::scpA(E52C, H235C), dnaN(N114C, V313C)::specR, parB(N112S)::kanR, trpC2* |
| BSG2058 | *1A700, smc-TEV-HaloTag ftsY::tetL, parB-mGFPmut1::ermB, fabG::specR::tos-rtp, trpC2* |
| BSG680 | *168 ED, CAT::scpA(E52C, H235C), smc(S19C, R558C, N634C, R1032C) ftsY::ermB, trpC2* |
| BSG1991 | *168 ED, CAT::scpA(E52C, H235C), smc(S19C, R558C, N634C, R1032C) ftsY:ermB, ΔparB::kanR, trpC2* |
| BSG1995 | *168 ED, CAT::scpA(E52C, H235C), smc(S19C, R558C, N634C, R1032C, E1118Q) ftsY::ermB, trpC2* |

Domínguez-Cuevas, P., Mercier, R., Leaver, M., Kawai, Y., and Errington, J. 2012. The rod to L-form transition of Bacillus subtilis is limited by a requirement for the protoplast to escape from the cell wall sacculus. *Mol. Microbiol.* **83**:52–66. doi:10.1111/j.1365-2958.2011.07920.x.
